# Alveolar Soft Part Sarcoma Metastatic to Small Bowel Mucosa Causing Polyposis and Intussuseption

**DOI:** 10.1080/13577140120048584

**Published:** 2001-09

**Authors:** Michael S. Sabel, John F. Gibbs, Allan Litwin, Brian Mcgrath, William B. Kraybill, John J. Brooks

**Affiliations:** 1 Department of Surgical Oncology Roswell Park Cancer Institute and State University of Buffalo at New York Buffalo New York USA; 2 Department of Radiology Roswell Park Cancer Institute and State University of Buffalo at New York Buffalo New York USA; 3 Department of Pathology and Laboratory Medicine Roswell Park Cancer Institute and State University of Buffalo at New York Buffalo New York USA; 4 Division of Surgery Roswell Park Cancer Institute Elm and Carlton Streets Buffalo NY 14263 USA

## Abstract

A report of alveolar soft part sarcoma metastatic to the small bowel is presented. Hematogenous metastases to the small bowel from primary tumors outside the abdominal cavity are uncommon, and most remain asymptomatic and are not discovered until autopsy. However, small bowel metastases can lead to intestinal obstruction, intussuseption or even perforation. While metastases to the small bowel have been described for other tumor types, including melanoma and lung cancer, this is extremely uncommon for sarcoma, especially alveolar soft part sarcoma. We describe a 42-year-old male with a long history of alveolar soft part sarcoma, metastatic to the lung and brain, who developed an intussuseption from metastases to the small bowel.


*Sarcoma* (2001) **5**, 133–137

ORIGINAL ARTICLE

### Alveolar soft part sarcoma metastatic to small bowel mucosa causing polyposis and intussuseption

MICHAEL S. SABEL[1], JOHN F. GIBBS[1], ALLAN LITWIN[2], BRIAN MCGRATH[1], WILLIAM B. KRAYBILL[1] & JOHN J. BROOKS,[3]

*[1]Department of Surgical Oncology, [2]Department of Radiology, and [3]Department of Pathology and Laboratory Medicine, Roswell Park Cancer Institute and State University of Buffalo at New York, Buffalo, New York, USA*

**Abstract**

A report of alveolar soft part sarcoma metastatic to the small bowel is presented. Hematogenous metastases to the small bowel from primary tumors outside the abdominal cavity are uncommon, and most remain asymptomatic and are not discovered until autopsy. However, small bowel metastases can lead to intestinal obstruction, intussuseption or even perforation. While metastases to the small bowel have been described for other tumor types, including melanoma and lung cancer, this is extremely uncommon for sarcoma, especially alveolar soft part sarcoma. We describe a 42-year-old male with a long history of alveolar soft part sarcoma, metastatic to the lung and brain, who developed an intussuseption from metastases to the small bowel.

### Introduction

Alveolar soft part sarcoma (ASPS) is a rare tumor that comprises approximately 0.5–1.0% of all soft tissue sarcomas.[1] ASPS has an unusual biologic behavior and its histogenesis has been the impetus for a number of recent reports. Metastasis occurs in about 68% of cases and is primarily hematogenous; lymph node metastasis is rare, most often ASPS spreads to the lung, bone and brain.[2] However, despite the number of case reports in the literature, ASPS metastatic to the small bowel has only once been reported.[3] We describe here a patient with ASPS that metastasized to the small bowel, causing intussuseption due to mucosal polyposis.

### Case report

The patient is a 42-year-old male whose history dates back to October 1984. when he noted a mass in the left anterior thigh. A magnetic resonance imaging (MRI) scan demonstrated a $9 \times 6 \times 5$ cm$^3$ mass involving the left vastus lateralis, rectus femoris and vastus intermedius muscles. Tru-cut needle biopsy was consistent with low-grade sarcoma. Computed tomography (CT) scans of the chest and bone were both negative. The patient underwent definitive surgical resection with negative margins. Final pathology demonstrated ASPS. The patient had post-operative adjuvant radiation therapy; 56 Gy delivered in 28 fractions over 37 days, using a shrinking field technique.

The patient remained disease free until May 1995 when he was noticed on chest CT to have multiple small bilateral pulmonary metastases. At this time, the patient was asymptomatic and did not want further therapy. Follow-up CT scans demonstrated minimal progression of the pulmonary disease over the next 3 years. In July 1998, the patient had the onset of hemoptysis and right-sided pleuritic pain. Bronchoscopy demonstrated cancer in the bronchial tree. At this point, the patient was begun on chemotherapy consisting of Adriamycin, ifosfamide and MESNA. He tolerated this well and demonstrated a good response. However, in October 1998, the patient had worsening dyspnea and a dramatic decline in his exercise capacity. Echocardiogram showed a dilated right ventricle with a large clot in the right outflow tract. MRI scan demonstrated that this was not a clot, but rather a right ventricular tumor. The patient was anti-coagulated and managed medically with control of his heart failure. In July 1999, the patient developed visual changes and numbness in his right arm. Head CT demonstrated multiple metastatic brain lesions. He underwent craniotomy with resection of the two largest lesions. This was followed by gamma knife radiosur-

Correspondence to: John Gibbs, M.D., Division of Surgery, Roswell Park Cancer Institute, Elm and Carlton Streets, Buffalo, NY 14263, USA. Tel: (+1) 716 845 5807; Fax: (+1) 716 845 3434, E-mail: john.gibbs@roswellpark.org

1357–714X print/1369–1643 online/01/030133–05 © 2001 Taylor & Francis Ltd
DOI: 10.1080/13577140120071669

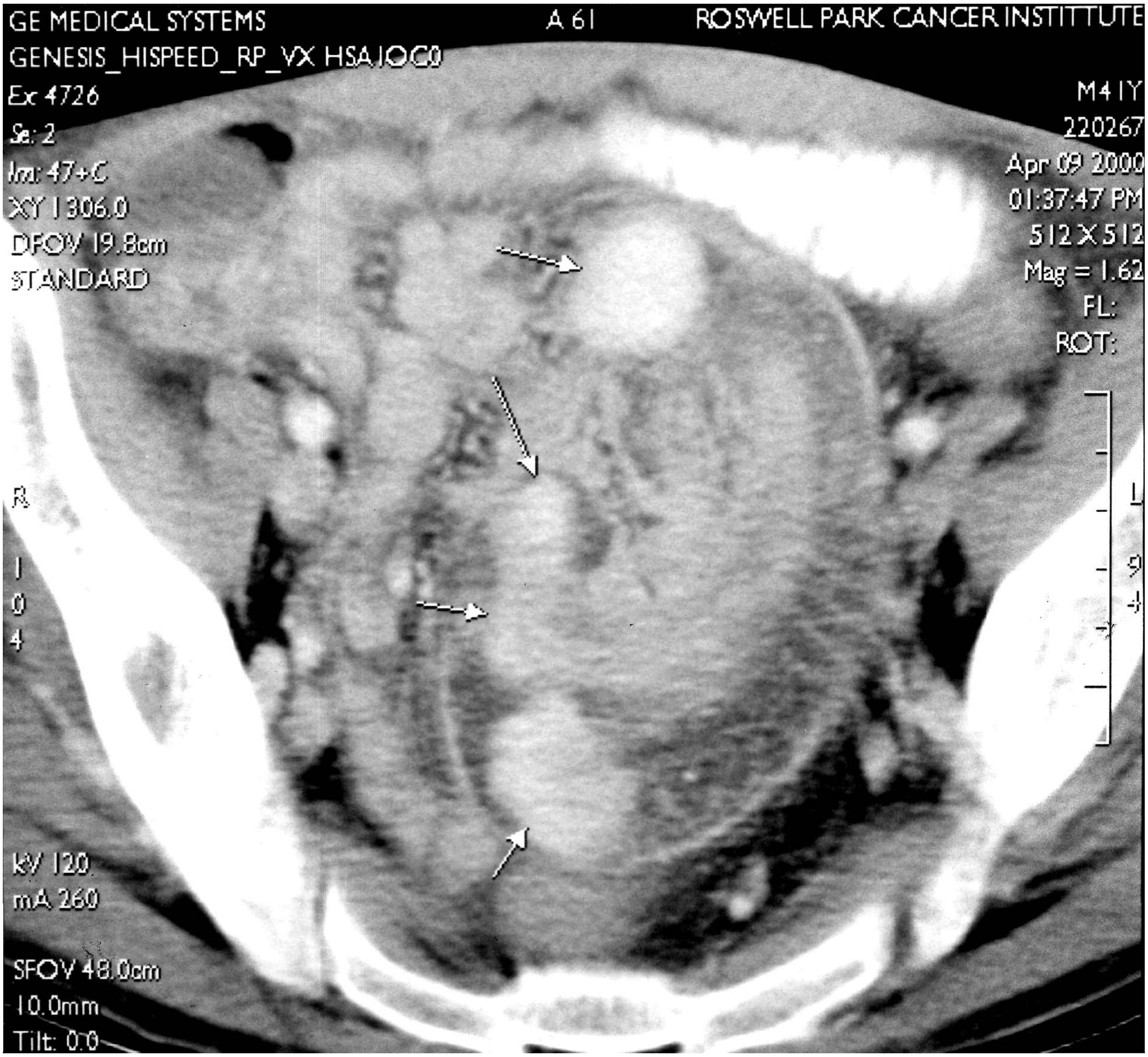

**Fig. 1.** *Image from a computed tomography scan of the abdomen and pelvis with oral and intravenous contrast. The image shows several hypervascular masses (arrows) within a small-bowel intussuseption with wall edema.*

gery to the smaller lesions. The patient was also treated with palliative radiation to the chest.

Despite a 5-year history of ASPS metastatic to the lung, mediastinum, heart, brain and liver, the patient was doing relatively well until April 2000 when he presented with complaints of lower quadrant abdominal pain. CT scan of the abdomen demonstrated an intussusception as well as multiple small bowel metastases (Fig. 1). He was brought to the operating room for exploration and underwent small bowel resection along with resection of several additional intra-abdominal sarcomas that appeared to be near-obstructing. Post-operatively, the patient did well and remains asymptomatic 15 years after the primary diagnosis and 5 years after the development of metastatic disease.

### Pathology

The small bowel resection was in two parts, with the larger 43 cm segment containing five sessile polypoid masses protruding from the mucosal surface. On section, these tumors were tan in color and varied in size from 1 to 3.5 cm in diameter. One of the larger polyps was the cause of the intussuseption (Fig. 2). All tumors were histologically identical and similar to the

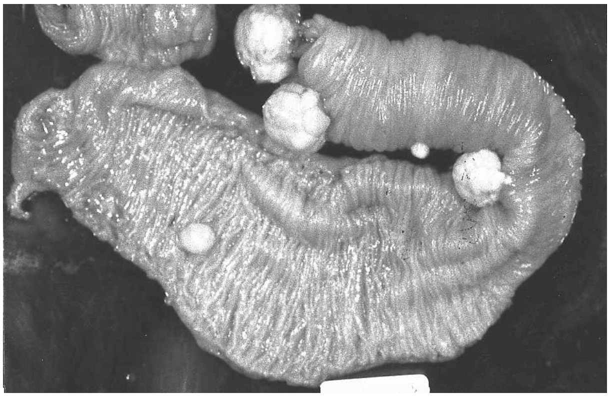

**Fig. 2.** *Small intestine with polypoid mucosal metastases from alveolar soft part sarcoma. Note the intusseptions polyp at the top center.*

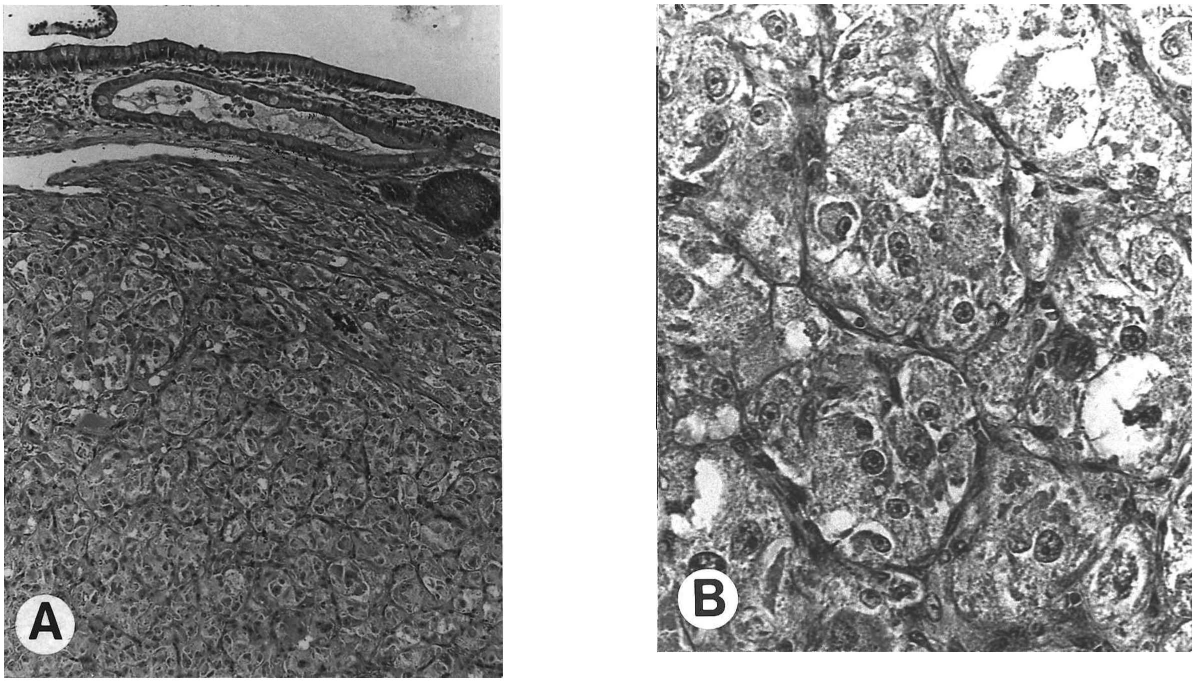

**Fig. 3.** *Alveolar soft part sarcoma. Tumor bulges into the lumen of the small bowel below the attenuated mucosa (A). At high power, round to polygonal cells with granular cytoplasm and oval nuclei form alveolar structures (B).*

primary tumor and other metastases. The alveolar appearance was apparent at low power, caused by the grouping of cells surrounded by thin blood vessels. The tumor cell cytoplasm was eosinophilic and granular, and the nuclei were uniform, large, vesicular, and contained prominent nucleoli (Fig. 2). Crystal-like rods were highlighted by a Periodic-Acid Schiff stain. Growth within the mucosa of the intestine was noted in all lesions; there was invasion into the submucosa, but the muscular layer was intact and no serosal tumor was seen. The other bowel segment exhibited a similar 1 cm. mucosal tumor.

### Discussion

Hematogenous metastases to the gastrointestinal tract from primary tumors outside the abdominal cavity are uncommon, but not rare. Most of these cases remain asymptomatic and are not discovered until autopsy.[4] However, small-bowel metastases can lead to intestinal obstruction, intussuseption or even perforation. Metastases to the small bowel have most commonly been described from melanoma[5,6] and from lung cancer,[7–11] but it as also been described as originating from cancers of the larynx,[12] thyroid,[13] and breast.[14] While sarcoma metastasizing to the small bowel has been described,[15–17] this is an extremely rare occurrence, and has only once been described for ASPS.[3]

ASPS was described initially in 1952 by Christopherson *et al.*[18] Over the next 50 years, the literature has been replete with articles debating the histogenesis of this tumor. When first described, the authors considered the possibilities that ASPS could represent metastatic renal cell carcinoma, endothelioma, liposarcoma, rhabdomyosarcoma, paraganglioma, or malignant granular-cell 'myoblastoma'. As numerous ultrastructural studies were performed, there developed increasing support that ASPS had a myogenous derivation. The presence of secretory-like granules in ASPS, similar to those in the carotid body, suggested that the histogenesis was related to paragangliomas. With the advent of immunohistochemistry, attempts to define the histogenesis using a broad immunohistochemical panel has added to the controversy.[2,19] ASPS fails to express neurofilament, met-enkaphalin, leu-enkaphalin and neuron-specific enolase as would be expected with a paraganglioma. However, ASPS also does not express desmin and myoglobin, which argues against a myogenic origin. Other authors have found immunoreactivity for fairly specific myogenic markers;[20–23] however, there is presently no conclusive evidence that it represents a unique type of muscle-derived tumor.[24] Almost 50 years after the initial description, the histogenesis remains a mystery.

The case presented in the present study demonstrates many of the typical features of ASPS. Clinically, ASPS tends to occur in younger patients, with a median age of 30 in males and 20 in females.[25] ASPS most often originates in the buttock or thigh in adults, while in children it usually originates in the head and neck region.[26] The tumor is characterized by slow growth and an early high frequency of distant metastases, with a proclivity for metastases to the lung, bone and brain. While the presence of metastases at the time of diagnosis carries a poorer prognosis (median survival time of 3 years), early metastases did not preclude a long survival time.[2] One unusual aspect to this patient is that, despite the widespread metastatic disease and presence of the highly unusual small-bowel metastases, this patient has not developed bone or subcutaneous metastases, both of which are common with ASPS.

The patient described in this report has a 15-year survival from the time of his initial diagnosis to this most recent episode, and a 5-year survival from the onset of metastases. After multiple therapeutic interventions and despite the decision to observe rather than treat the pulmonary metastases, he is still alive and presently asymptomatic. There are several reports in the literature of long-term survivors with metastatic ASPS. One case report describes a patient who developed the primary chest wall lesion in 1960, pulmonary metastases in 1981, and brain and renal metastases in 1992. The patient was still alive at the time with surgical resections only.[27] Another series describes a patient who underwent excision of 130 pulmonary and three brain metastases during four thoracotomies and two craniotomies, and was still alive 98 months after excision of the primary lesion.[28] These reports and others[29–31] suggest that the aggressive and repeated treatment of metastatic disease in ASPS patients may influence long-term survival and maintenance of good performance status in patients.

In conclusion, we describe the unreported occurrence of a sarcomatous metastasis to small-bowel mucosa causing the unusual features of polyposis and intussuseption in a patient with a very rare tumor, namely ASPS. As this case demonstrates, surgical intervention in this particular sarcoma type adds significantly to patient survival despite long-standing metastatic disease.

### References

1 Enzinger FM, Weiss SW. Alveolar soft part sarcoma. *Soft Tissue Tumors*, 3rd edition, vol. 13. St. Louis, MO: CV Mosby, 1995:1067–1074.
2 Lieberman PH, Brennan MF, Kimmel M, *et al.* Alveolar soft-part sarcoma: a clinicopathologic study of half a century. *Cancer* 1989; 63:1–13.
3 Sueyoshi T, Kanetuki I, Nishi H, Ohshiro K, Hori A. A case of gastrointestinal bleeding from metastatic alveolar soft part sarcoma of the jejunum. *Nippon Igaku Hoshasen Gakkai Zasshi* 1996; 56:712–714.
4 Avagnina A, Elsner B, DeMarco L, *et al.* Pulmonary rhabdomyosarcoma with isolated small bowel metastasis. A report of a case with immunohistochemical and ultrastructural studies. *Cancer* 1984; 53:1948–1951.
5 Krige JE, Nel PN, Hudson DA. Surgical treatment of metastatic melanoma of the small bowel. *Am Surg* 1996; 62:658–663.
6 Rintegen DS, Thompson W, Garbutt J, Seigler HF. Radiologic, endoscopic and surgical considerations of malignant melanoma metastatic to the small intestine. *Curr Surg* 1984; 418:87–89.
7 Berger A, Cellier C, Daniel C, *et al.* Small bowel metastases from primary carcinoma of the lung: clinical findings and outcome. *Am J Gastroenterol* 1999; 94:1884–1887.
8 Dalton ML, Simon KB, Gatling RR, Koury AM. Large cell carcinoma of the lung with isolated jejunal metastasis. *J Mississippi State Med Assoc* 1989; 30:361–363.
9 Quayle AR, Holt S, Clark RG. Jejunal perforation secondary to metastatic bronchogenic carcinoma. *Postgrad Med J* 1985; 61:163–165.
10 McNeill PM, Wagman LD, Neifeld JP. Small bowel metastases from primary carcinoma of the lung. *Cancer* 1987; 59:1486–1489.
11 Stenbygaard LE, Sorensen JB. Small bowel metastases in non-small cell lung cancer. *Lung Cancer* 1999; 26:95–101.
12 Airoldi M, Gabriele P, Succo G, Valente G, Brando V. Small bowel metastasis from squamous cell carcinoma of the larynx. A case report. *Tumori* 1993; 79:286–287.
13 Phillips DL, Benner KG, Keeffe EB, Traweek ST. Isolated metastasis to small bowel from anaplastic thyroid carcinoma. With a review of extra-abdominal malignancies that spread to the bowel. *J Clin Gastroenterol* 1987; 9:563–567.
14 Fraser-Moodi A, Burn I. Small-bowel metastases from carcinoma of the breast. *Proc R Soc Med* 1974; 67:1023–1024.
15 Gerst PH, Levy J, Swaminathan K, Kshettry V, Albu E. Metastatic leiomyosarcoma of the uterus: unusual presentation of a case with late endobronchial and small bowel metastases. *Gynecol Oncol* 1993; 49:271–275.
16 Avagnina A, Elsner B, DeMarco L, *et al.* Pulmonary rhabdomyosarcoma with isolated small bowel metastasis. A report of a case with immunohistochemical and ultrastructural studies. *Cancer* 1984; 53:1948–1951.
17 Panizo-Santos A, Sola I, Lozano MD, de Alava E, Pardo J. Metastatic osteosarcoma presenting as a small bowel polyp. A case report and review of the literature. *Arch Pathol Lab Med* 2000; 124:1682–1684.
18 Christopherson WM, Foote Jr FW, Stewart FW. Alveolar soft part sarcoma: structurally characteristic tumors of uncertain histogenesis. *Cancer* 1952; 5:100–111.
19 Auerbach HE, Brooks JJ. Alveolar soft part sarcoma: a clinicopathologic and immunohistochemical study. *Cancer* 1987; 60:66–73.
20 Ordonez NG, Jae YR, Mackay B. Alveolar soft part sarcoma: an ultrastructural and immunocytochemical investigation of its histogenesis. *Cancer* 1989; 63:1721–1736.
21 Hirose I, Kudo E, Haseqawa T, *et al.* Cytoskeletal properties of alveolar soft part sarcoma. *Hum Pathol* 1990; 21:204–211.
22 Miettinen M, Ekfors T. Alveolar soft part sarcoma. Immunohistochemical evidence for muscle cell differentiation. *Am J Clin Pathol* 1990; 93:32–38.
23 Yamaguchi K, Soejima J, Maeda S, *et al.* Alveolar soft part sarcoma. A case report with immunohistochemical study. *Jpn Surg* 1990; 20:476–480.
24 Ordonez NG. Alveolar soft part sarcoma: a review and update. Advances in *Anatomic Pathol* 1999; 6:125–139.
25 Lieberman PH, Foote FW, Stewart FW, Berg JW. Alveolar soft part sarcoma. *JAMA* 1966; 198:121–125.
26 Matsuno Y, Mukai K, Itahashi M, *et al.* Alveolar soft part sarcoma. A clinicopathologic and immunohistochemical study of 12 cases. *Acta Pathol Jpn* 1990; 40:199–205.
27 Lillehei KO, Kleinschmidt-DeMasters B, Mitchell DH. Alveolar soft part sarcoma: an unusually long interval between presentation and brain metastases. *Hum Pathol* 1993; 24:1030–1034.

28 Kodoma K, Doi O, Higashiyama M, *et al.* Surgery for multiple lung metastases from alveolar soft part sarcoma. *Surg Today* 1997; 27:806–811.
29 Wang CH, Lee N, Lee LS. Successful treatment for solitary brain metastasis from alveolar soft part sarcoma. *J Neuro-Oncol* 1995; 25:161–166.
30 Bindal RK, Sawaya RE, Leavens ME, Taylor SH, Guinee VF. Sarcoma metastatic to the brain: results of surgical treatment. *Neurosurgery* 1994; 35:185–190.
31 Ueda T, Uchida A, Kodama K, *et al.* Aggressive pulmonary metastaasectomy for soft tissue sarcomas. *Cancer* 1993; 72:1919–1925.
